# Supplementary material for: Association between antenatal corticosteroid use and perinatal mortality among preterm births in hospitals in Tanzania
Source: PLoS One. 2021 Jul 22;16(7):e0254916. doi: 10.1371/journal.pone.0254916 (PMC8297919; doi:10.1371/journal.pone.0254916)
Supplement: Attachment — Submitted filename: Response to reviewers.docx [file pone.0254916.s002.docx]

PONE-D-21-04378
Association between antenatal corticosteroid use and perinatal mortality among preterm births in hospitals in Tanzania.

Dear Editor

Thank you for your kind review and for considering our manuscript for publication in PLOS ONE. Also, we thank the reviewer for his generous comments on the manuscript and we have edited the manuscript to address his concerns.

We have revised the whole manuscript to meet PLOS ONE's style requirements. Furthermore, we have rephrased the ethics statement to include additional information as requested. We have also responded to each of the reviewers’ comments as shown in the table below.

**Response to reviewer comments**

| **S/N** | **Comment** | **Response** | **Page number** |
| --- | --- | --- | --- |
| 1 | I would suggest the authors update the background to strengthen the rationale for their cohort study. I would discuss the Althabe study, the ACTION trial and the updated Cochrane antenatal corticosteroid review in more detail (reference 22 has been updated). In addition the authors have not mentioned the Cochrane review entitled; Strategies for optimising antenatal corticosteroid administration for women with anticipated preterm birth which is extremely pertinent to their trial | We have significantly expanded, strengthened and updated the background of the study. The finding of the 2017 Cochrane review, the Althabe study the WHO action trial are discussed in more detail as it the findings of the recently published Cochrane review entitled “Strategies for optimising antenatal corticosteroid administration for women with anticipated preterm birth” | 5-6 |
| 2 | Line 107: The authors definition of stillbirth. Is this not neonatal death? | We have modified the definition (Line 167):  “For this study, stillbirth was defined as the death or loss of baby after 20 weeks of pregnancy that occurred before or during delivery. The baby had an Apgar score of 0 at both 1 and 5 minutes after delivery.” | 7 |
| 3 | Line 127: I would rephrase 'explanatory variables' perhaps to baseline demographics and associated factors | Line 183: As suggested, we have rephrased “explanatory variables” to “baseline demographics and associated factors were included” | 8 |
| 4 | Lines 127-128: ACS should not be included among the explanatory variables | ACS was removed from the explanatory variables. | 8 |
| 5 | **Data analysis:** Line 142: The authors mention apgar scores and neonatal sepsis-this is not covered in the initial aims outlined in the study. There needs to be consistency. The authors need to distinguish between study variables and demographics of the population and consider this when undertaking analysis about antenatal corticosteroid use. The authors need to be careful that any analysis undertaken is clinically meaningful/relevant rather than data mining | We have revised the abstract as follows to state the aims of the study:  The primary aims of this study were to investigate if exposure to antenatal corticosteroids (ACS) was associated with lower rates of perinatal mortality (primary outcome) and other adverse perinatal outcomes (i.e., stillbirth, early neonatal mortality, APGAR score of < 7 at 5 mins, neonatal sepsis and respiratory distress syndrome) in preterm infants in hospitals in Tanzania. We also examine factors associated with administration of ACS among women at risk of preterm delivery.  We have also added the following to the Introduction:  This study investigated if exposure to ACS was associated with lower rates of perinatal mortality (primary outcome) and other perinatal outcomes (stillbirth, early neonatal mortality, APGAR score of < 7 at 5 mins, neonatal sepsis and RDS) in preterm infants. We also investigated factors associated with administration of ACS to women at risk preterm deliveries in district, regional and tertiary hospitals in Nyamagana and Sengerema districts in Tanzania.  We have also added the following in the study design  The primary outcome, perinatal mortality, was defined as stillbirth or early neonatal mortality before day 7. Secondary outcomes were stillbirth, early neonatal mortality, APGAR score at 5 mins, neonatal sepsis and RDS.  We have rephrased the data analysis section to:  Differences in perinatal outcomes between infants exposed to ACS and those who were not were examined using fishers exact test and chi-square tests where appropriate.  Analysis about antenatal corticosteroid use have been described as follows:  Multivariate regression analyses were performed to examine the effects of administration of ACS on perinatal outcomes, controlling for factors with significant associations (gestational age, birth weight, level of health facility, multiple pregnancy and delivery mode). | 2  6  8  8  9 |
| 6 | **Results**  Line 209: The authors primary aim relates to perinatal mortality-the authors report on apgar scores, neonatal sepsis and RDS. This is not specified in the initial methods | We have included APGAR scores, neonatal sepsis and RDS in the aims, methods and statistical analysis. See our responses to reviewer comment 5. | 2, 6,8 & 9 |
| 7 | I think the authors should review the Tables-Table 3 and Table 4 are not particularly reader friendly | We have revised tables 3 and 4 to make them more reader friendly format | 12 -14 |
| 8 | Line 259: Gestational age should not be included in this table | We have removed gestational age from table 4 | 13 |
| 9 | **Discussion** Overall I think the discussion section need to be strengthened. The authors also need to discuss the WHO recommended criteria prior to antenatal corticosteroid administration. As one of the criterion is that gestational age assessment needs to be accurately undertaken and that there is no clinical evidence of maternal infection. I would imagine this might impact on administration of antenatal corticosteroids so should be discussed as an explanation for their results | We included the following:  The WHO recommends ACS for women at risk of preterm birth from 24 weeks to 34 weeks’ gestation in settings where the following criteria are met: gestational age assessment can be accurately undertaken, preterm birth is considered imminent, there is no clinical evidence of maternal infection, adequate childbirth care is available and the preterm newborn can receive adequate care if needed (including resuscitation, thermal care, feeding support, infection, treatment and safe oxygen use)  The present study included clinical settings with diverse neonatal care resources, from primary to tertiary level hospitals, that may have had differing methods and criteria for assessing eligibility for ACS treatment; gestational age was determined by either maternal self-reports of last menstrual period, measurement of fundal height or ultrasonographic examination. In contrast, the WHO ACTION trial was undertaken in hospital settings where the WHO criteria for ACS treatment could be met; gestational age determined by ultrasonographic examination. Also, in the WHO ACTION trial, women with maternal infection were excluded; however, in the present study, 8.15% of women who were administered ACS had maternal infection. | 15 &16 |
| 10 | Line 277/278: This Cochrane review has been updated and includes the action study. This paragraph needs to be changed and rephrased to reflect the new evidence/conclusions. | We have rephrased to:  This finding is consistent with a recently updated Cochrane review of randomized controlled trials conducted in hospital settings, which reported a lower rate of perinatal mortality regardless of resource setting among infants who were exposed to ACS (RR 0.85, 95% CI 0.77 -0.93; 14 studies, 9833 infants). | 14 |
| 11 | Line 282: I do not think the references used are the most appropriate given the recently published studies | We have removed outdated references and added the following in discussion:  Our findings, however, were not consistent with those of the ACT trial, which reported no difference in perinatal mortality between the ACS exposed and unexposed groups. This could be attributable to differences in health care settings and differences in maternal and perinatal baseline characteristics. The ACT trial reported on deliveries from all levels of care, including primary health care and care at the community level. In contrast, the present study reported on only hospital deliveries at district, regional and zonal hospitals. | 15 |
| 12 | Lines 295/296: I think this needs to be rephrased the ACTION trial was a randomised control trial including over 2852 women and 3070 fetuses. | We have rephrased to:  For example, the WHO ACTION trial was a randomised control trial conducted in 29 secondary- and tertiary-level hospitals across Bangladesh, India, Kenya, Nigeria, and Pakistan, whereas the present study was a prospective observational chart review study conducted only in Tanzania. | 15 & 16 |
| 13 | The authors need to be a little careful not to overreach and there should be some mention regarding the limitations of their study as it is not a randomised trial and the inherent biases associated with cohort studies. | We included the following:  However, our study has some limitations. First, it was a prospective observational study. Thus, the inherent biases associated with observational studies (e.g., selection bias, information bias, confounding) could have influenced our outcomes. We attempted to control for selection bias by including all stillbirths and live births of preterm infants delivered at the selected hospitals between 24 weeks 0 days to 34 weeks 6 days of gestation between July 2019 to February 2020. Informational bias was controlled for by using a standard data collection form and confounding by considering a number of factors that could be associated with exposure to ACS and pregnancy outcomes. | 17 |
| 14 | There are a few typographical errors. Lines: 250, 300 | We have corrected typographical errors. (Line: 280, 341) | 13&16 |

We look forward to hearing from you regarding our submission. We would be very happy to respond to any further questions and comments that you may have to make our manuscript suitable for publication.

Kind regards, and on behalf of the authors,

STANLEY MWITA, BPHARM, MSc
